# Impact of Hashimoto Thyroiditis on Long-Term Outcomes in Differentiated Thyroid Carcinoma

**DOI:** 10.3390/cancers18121938

**Published:** 2026-06-14

**Authors:** Jasna Mihailović, Ivana Starčević, Slađana Novković-Ostojić, Tijana Vasiljević, Nataša Prvulović Bunović, Bojana Šćepanović

**Affiliations:** 1 Department of Nuclear Medicine and Molecular Imaging, Faculty of Medicine, University of Novi Sad, Hajduk Veljkova 3, 21000 Novi Sad, Serbia; ivana.starcevic@mf.uns.ac.rs (I.S.); natasa.prvulovic-bunovic@mf.uns.ac.rs (N.P.B.); 2 Division of Nuclear Medicine, Oncology Institute of Vojvodina, Put dr Goldmana 4, 21204 Sremska Kamenica, Serbia; 3 Radiology Centre, Institute for Pulmonary Diseases of Vojvodina, Put dr Goldmana 4, 21204 Sremska Kamenica, Serbia; 4 Department of Pathology, Faculty of Medicine, University of Novi Sad, Hajduk Veljkova 3, 21000 Novi Sad, Serbia; tijana.vasiljevic@mf.uns.ac.rs; 5 Department of Pathology and Laboratory Diagnostic, Oncology Institute of Vojvodina, Put dr Goldmana 4, 21204 Sremska Kamenica, Serbia; 6 Centre for Diagnostic Imaging, Oncology Institute of Vojvodina, Put dr Goldmana 4, 21204 Sremska Kamenica, Serbia; scepanovic.bojana@onk.ns.ac.rs

**Keywords:** hashimoto thyroiditis, differentiated thyroid carcinoma, outcome, long-term

## Abstract

Hashimoto thyroiditis (HT) coexists with differentiated thyroid carcinoma (DTC), particularly papillary thyroid carcinoma, in approximately 25% of cases. However, the impact of this association on DTC outcomes remains controversial. The aim of this study was to analyze the influence of Hashimoto thyroiditis on disease-specific survival (DSS) and recurrence-free survival (RFS) in DTC patients. **Methods**: A retrospective study conducted at our institution between 2007 and 2020 analyzed 707 DTC patients treated with surgery and/or I-131 therapy. Cox proportional hazards regression was used to identify independent predictors, including sex, age, tumor histology, HT status, and initial TNM stage. **Results**: Among 707 DTC patients, 628 (88.8%) had papillary cancer, 582 (82.3%) were female, 395 (55.9%) were <55 years old; HT coexisted in 137 (19.4%) patients. During follow-up, 23 (3.25%) developed recurrent disease; at last follow-up, 638 (90.2%) were alive. Initial distant metastases (*p* < 0.001) and higher T stage (*p* = 0.002) independently predicted worse DSS. For RFS, male sex (*p* = 0.015), higher T stage (*p* = 0.018), and lymph node involvement (*p* = 0.023) independently predicted an increased risk of recurrence. HT was not an independent predictor of DSS (HR 0.97, 95% CI 0.21–4.52; *p* = 0.964) or recurrence (HR 0.36, 95% CI 0.05–2.73; *p* = 0.322). **Conclusions**: Although Hashimoto thyroiditis was associated with favorable clinicopathological features, it was not independently associated with disease-specific or recurrence-free survival. Conventional staging parameters, particularly tumor stage, remain the principal determinants of prognosis in differentiated thyroid cancer.

## Simple Summary

Hashimoto thyroiditis (HT) is frequently found in patients with differentiated thyroid carcinoma (DTC), especially papillary thyroid cancer, but its influence on long-term outcomes remains unclear. In this retrospective study, we analyzed 707 patients with DTC treated at our institution between 2007 and 2020 to evaluate the impact of HT on recurrence-free survival and disease-specific survival. Patients with coexisting HT showed lower recurrence rates and generally more favorable outcomes compared with patients without autoimmune thyroid disease. However, after adjustment for established prognostic factors, HT was not identified as an independent predictor of recurrence or survival. Tumor stage, particularly T and M stage, remained the strongest determinant of prognosis. These findings suggest that the possible protective effect of HT may be limited and that conventional clinicopathological factors continue to play the central role in risk assessment and patient management in DTC.

## 1. Introduction

Thyroid cancer is the most common endocrine malignancy, accounting for approximately 3.4% of all cancers diagnosed worldwide each year [1,2]. The majority of thyroid tumors arise from thyroid follicular epithelial cells. Differentiated thyroid cancers (DTCs) are follicular cell–derived neoplasms and include two main histological types: papillary thyroid cancer (PTC) and follicular thyroid cancer (FTC). These tumors are generally indolent and are associated with a favorable prognosis, with 10-year survival rates of up to 90% [3,4]. According to the GLOBOCAN (Global Cancer Statistics) report covering 36 cancers across 185 countries, an estimated 821,173 new cases of thyroid cancer (4.1% of all sites) were diagnosed worldwide, making it the seventh most commonly diagnosed cancer worldwide. Thyroid cancer-related mortality accounted for 47,485 (0.5% of all cancer deaths) in both sexes, ranking it 24th among all cancers globally [5]. In the United States, according to the American Cancer Society, an estimated 45,240 new cases of thyroid cancer are expected in 2026 (32,000 in women and 13,240 in men), with approximately 2320 deaths (1220 in women and 1100 in men) [6].

Chronic lymphocytic thyroiditis (i.e., Hashimoto thyroiditis) is the most common autoimmune disease and mostly occurs in women aged 30–50, although it can also affect the juvenile population. It is the most common cause of hypothyroidism in iodine-sufficient regions and is characterized by thyrocyte destruction resulting in decreased hormone production [7,8,9,10]. Hashimoto thyroiditis (HT) was named after the Japanese surgeon Hakaru Hashimoto, who first described lymphocytic infiltration of the thyroid gland in 1912 and termed it *Struma lymphomatosa* [11]. HT shows specific autoantibodies, including thyroid peroxidase (TPOAb) and thyroglobulin antibodies (TgAb), along with lymphocytic infiltration of the thyroid gland [7,8]. The discovery of these autoantibodies associated with HT in 1936 led several researchers to recognize HT as a prototypical example of an autoimmune disease characterized by tissue destruction [12,13].

The coexistence of HT and differentiated thyroid carcinoma, particularly papillary thyroid carcinoma, has been widely reported in the literature, suggesting a potential biological link between chronic autoimmune inflammation and thyroid tumorigenesis [14,15,16,17,18].

The mechanisms and clinical significance of this association remain controversial and are supported by limited evidence, particularly with respect to long-term survival outcomes. While some authors suggest that concomitant HT may be associated with more favorable outcomes and lower risk of recurrence [14,15,16], others have reported no significant differences in disease-specific survival or long-term recurrence rates between patients with and without HT [17]. These literature inconsistencies imply that the prognostic impact of concomitant HT in DTC patients remains to be definitively established.

This study aims to evaluate the influence of HT on disease-specific survival (DSS) and recurrence-free survival (RFS) in patients with DTC.

## 2. Materials and Methods

### 2.1. Patients

This retrospective single-center study was conducted at the Oncology Institute of Vojvodina between January 2007 and December 2020. During this period, 4659 patients with thyroid nodules underwent surgical treatment. Final histopathological examination revealed benign disease in 3849 patients (82.6%), while 810 patients (17.4%) were diagnosed with malignant tumors. Histopathological diagnosis of DTC was confirmed in 747 patients (16%). Among these, 40/747 patients (5.4%) were excluded due to a history of previously diagnosed non-thyroid malignancy. An additional 41 patients (0.9% of the total cohort) had other thyroid carcinoma subtypes, including medullary (*n* = 32; 0.7%), anaplastic (*n* = 2; 0.04%), and poorly differentiated carcinoma (*n* = 7; 0.15%). Furthermore, in 22 patients (0.5% of the total cohort), the thyroid lesion represented a metastasis from another primary malignancy. Ultimately, 707 patients with DTC were included in the study.

The inclusion criteria were as follows: (a) surgery performed at our institution; (b) histopathological diagnosis of differentiated thyroid carcinoma, including papillary, follicular, and oncocytic carcinoma and their subtypes; (c) perioperative measurement of thyroid autoantibodies; and (d) age ≥18 years. The exclusion criteria were: (a) concomitant non-thyroid malignancy; and (b) histological types other than DTC.

**Figure 1:**
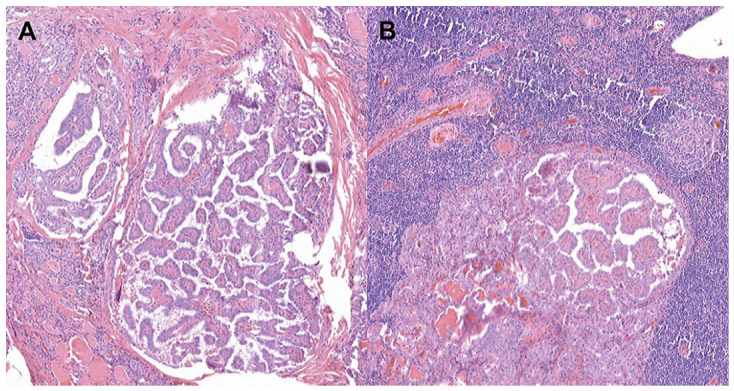
Papillary thyroid carcinoma with lymphocytic inflammatory infiltrate. (**A**). Classic papillary thyroid carcinoma with a scarce lymphocytic inflammatory infiltrate localized to the peritumoral region (H&E, 12.3×). (**B**). Diffuse lymphocytic infiltrate with prominent germinal centers surrounding the papillary thyroid carcinoma in a patient with Hashimoto thyroiditis. Adjacent tissue showing prominent atrophy, fibrosis, and oxyphilic metaplasia (H&E, 7.7×).

Patients were stratified into two groups according to the presence or absence of concomitant HT. The diagnosis of HT was established based on histopathological findings and/or the presence of thyroid autoantibodies (anti–thyroid peroxidase and/or anti-thyroglobulin antibodies). Histopathological confirmation required diffuse lymphoplasmacytic infiltration, the presence of oxyphilic cells, formation of lymphoid follicles with reactive germinal centers, and atrophic changes in the surrounding non-neoplastic thyroid tissue. These inflammatory changes had to be identified in non-neoplastic areas of the thyroid parenchyma, clearly separate from the site of DTC. Peritumoral lymphocytic (inflammatory) infiltration was not considered diagnostic of HT (Figure 1).

The study was approved by the Ethics Committee of the Oncology Institute of Vojvodina (approval number 4/24/3-4460/2-4) and was conducted in accordance with the Declaration of Helsinki. The requirement to obtain informed consent was waived.

### 2.2. Treatment

All patients were treated according to the institutional Tumor Board protocol for DTC. Initial treatment consisted of total or near-total thyroidectomy, with or without postoperative radioactive iodine (I-131) therapy.

#### 2.2.1. Surgery

Initial surgery was performed in all patients referred to our institution for thyroid nodules suspicious for malignancy, previously confirmed cervical metastases, or fine-needle aspiration cytology (FNAC) results classified as Bethesda category III–VI.

The extent of surgery was determined in accordance with the American Thyroid Association (ATA) guidelines and included total thyroidectomy, subtotal thyroidectomy (lobectomy or partial tumorectomy), and neck dissection when indicated [19]. At our institution, intraoperative frozen section analysis was routinely performed in all patients. Preoperative lymph node status was assessed by ultrasound (US), with computed tomography (CT) performed when US findings were suspicious. Definitive nodal involvement was confirmed by histopathological examination. Tumors were classified according to the Tumor-Node-Metastasis (TNM) system, and stage grouping was assigned according to the 8th edition of the AJCC Cancer Staging Manual [20].

#### 2.2.2. Therapy with I-131

Low-risk DTC patients without nodal or distant metastases and without adverse histopathological features were not considered candidates for radioiodine ablation, unless specifically requested by the patient. The administered activity of radioiodine therapy was determined as follows: 3.7 GBq (100 mCi) of I-131 was used for ablation in patients without nodal or distant metastases (N0M0) and 5.55 GBq (150 mCi) in patients with regional or distant metastases (N1M0/N1M1).

Additional radioiodine therapy was administered in cases of unsuccessful ablation, persistent disease (defined as persistently detectable thyroglobulin [Tg] levels during L-thyroxine therapy), or recurrence detected on imaging. Post-therapy I-131 whole-body scintigraphy (WBS) was performed 72–96 h after radioiodine treatment in all patients. Following initial treatment, all patients received L-thyroxine therapy according to the ATA guidelines.

### 2.3. Laboratory Analyses

Analyses included routine measurements of Tg, TgAb, TPO-Ab, free triiodothyronine (FT3), free thyroxine (FT4), and thyroid-stimulating hormone (TSH). Measurements were performed using an electrochemiluminescence immunoassay (Roche Diagnostics GmbH, Mannheim, Germany). TgAb and TPO-Ab levels were considered positive if they exceeded 115 IU/mL and 34 IU/mL, respectively. Preoperative antithyroid antibody levels were assessed whenever data were available. However, not all measurements were performed at our institution. A proportion of patients were referred to our center for surgery and subsequent I-131 therapy after having completed their diagnostic work-up and laboratory analyses at other institutions. As a result, antithyroid antibody data were unavailable for that subset of patients. Due to incomplete data availability, we were unable to perform a reliable statistical analysis of the association between antithyroid antibody status and disease-free survival (DFS) or DSS.

### 2.4. Follow-Up and Outcome

Patients were regularly monitored every 3 months during the first year, every 6 months for the subsequent 5 years, and annually thereafter. Follow-up included clinical examination, neck ultrasonography, and laboratory testing.

Our patients underwent re-stratification 1 year after the initial therapy, based on their response to treatment. According to the ATA guidelines criteria, treatment outcomes were categorized into four distinct response groups. An excellent response was defined as the complete absence of clinical, biochemical, and structural evidence of disease, including negative imaging findings and low Tg levels (suppressed, Tg < 0.2 ng/mL or stimulated Tg < 1 ng/mL). A biochemical incomplete response is characterized by abnormal Tg levels (suppressed Tg > 1 ng/mL or stimulated Tg > 10 ng/mL) or increasing TgAb titers in the absence of identifiable structural disease (i.e., negative imaging findings). A structurally incomplete response included persistent or newly identified locoregional or distant metastases (any Tg value). An indeterminate response was characterized by nonspecific biochemical findings—such as suppressed Tg levels of 0.2–1 ng/mL, stimulated Tg 1–10 ng/mL, or stable/decreasing TgAb levels—or structural findings that could not be definitively classified as benign or malignant (e.g., indeterminate imaging results and/or faint uptake on I-131 WBS) [19].

Recurrent disease was defined as new evidence of locoregional disease or distant metastases occurring after at least 12 months of complete remission following initial treatment (i.e., disease-free status). Patients classified as having recurrent neck disease were diagnosed by ultrasound examination and fine-needle aspiration biopsy with cytological confirmation. Those with confirmed relapse underwent additional surgery (tumor excision or lymphadenectomy) or further RAI therapy (5.55 GBq). In patients with partial response or progressive pulmonary disease, additional RAI therapy was administered as indicated during follow-up. Whole-body or blood dosimetry was not performed for any RAI treatment administered for persistent or recurrent disease.

### 2.5. Statistical Analysis

The records of all 707 patients with DTC were retrospectively analyzed. Clinical and pathological data were systematically retrieved from institutional medical records. All relevant clinical information, including clinical presentation at diagnosis, detailed histopathological characteristics of the primary tumor (tumor size and histological subtype), and documented cause of death, was collected. Comprehensive longitudinal data on disease course were available for all patients throughout the follow-up period, enabling complete assessment of clinical outcomes.

The predefined study endpoints were the date of last follow-up and the date of death. Disease-specific survival and RFS were analyzed using Kaplan–Meier curves and compared by using the log-rank statistics. DSS was defined as the time from diagnosis to disease-specific death, with patients censored if alive or dead from other causes. RFS was defined as the time to structural recurrence or progression. In Cox proportional hazards regression models, covariates included age (<55 years vs. ≥55 years), sex (females vs. males), initial T stage (pT1–2 vs. pT3–4), initial lymph node status (N0 vs. N1), presence of distant metastases at presentation (M0 vs. M1), histological type of the tumor (papillary vs. follicular vs. oncocytic carcinoma), and HT status (DTC-HT vs. DTC-non-HT). The proportional hazards assumption was assessed using log-minus-log plots and time-dependent covariates. Statistical analyses were conducted using IBM SPSS Statistics for Windows, Version 20.0 (IBM Corp., Armonk, NY, USA). A *p*-value of <0.05 was considered statistically significant.

## 3. Results

Demographic and histopathological characteristics of DTC patients are presented in Table 1.

Among 707 patients, 628 (88.8%) had papillary carcinomas, 60 (8.5%) had follicular carcinomas, and 19 (2.7%) had oncocytic carcinomas. The cohort included 582 (82.3%) female patients (mean age 50.9 ± 13.8 years; range 19–84) and 125 (17.7%) male patients (mean age 52.0 ± 14.2 years; range 18–80). Overall, 395 (55.9%) patients were aged <55 years, and 312 (44.1%) were aged ≥55 years. According to the presence of HT, patients were stratified into two groups: 137 (19.4%) with concomitant HT (DTC-HT) and 570 (80.6%) without HT (DTC-non-HT).

Compared with the DTC-non-HT group, the DTC-HT group showed a significantly higher proportion of female patients (90.5% vs. 80.4%, *p* = 0.007), a higher frequency of low initial tumor stage (pT1–pT2) (86.2% vs. 76.1%, *p* = 0.015), and a lower rate of lymph node involvement at presentation (N1) (8.8% vs. 16.0%, *p* = 0.044).

The prevalence of distant metastases at presentation did not differ between the groups (2.2% vs. 2.8%, *p* = 0.915). Papillary carcinoma was more frequent in the DTC-HT group compared with the DTC-non-HT group (93.4% vs. 87.7%); however, this difference did not reach statistical significance (*p* = 0.079). Tumor multifocality did not differ between the DTC-HT and DTC-non-HT groups (30.7% vs. 28.4%, *p* = 0.672). Age at diagnosis also did not differ significantly between the groups (*p* = 0.342).

In the DTC-HT group, total thyroidectomy followed by radioiodine therapy (TT + RAI) was performed in 95 patients (69.3%), while total thyroidectomy alone (TT − RAI) was performed in 39 patients (28.5%). Subtotal thyroidectomy followed by radioiodine therapy was performed in 3 patients (2.2%), whereas no patients underwent subtotal thyroidectomy alone. In the DTC-non-HT group, total thyroidectomy followed by radioiodine therapy was performed in 397 patients (69.6%), and total thyroidectomy alone in 147 patients (25.8%). Subtotal thyroidectomy followed by radioiodine therapy (STT + RAI) was performed in 21 patients (3.7%), while subtotal thyroidectomy alone (STT − RAI) was performed in 5 patients (0.9%). There was no statistically significant difference in initial treatment between the analyzed groups (*p* = 0.526).

### 3.1. Follow-Up

All patients underwent long-term follow-up. For overall survival analysis, the mean and median follow-up durations were 112.9 and 109 months, respectively (range 1–222 months). Follow-up was slightly shorter in the DTC-HT group (mean 107.7 months, median 102 months, range 12–212 months) than in the DTC-non-HT group (mean 114.1 months, median 129 months, range 1–222 months).

Similar findings were observed for recurrence-free survival (RFS) analysis, for which the mean and median follow-up durations were 111.4 and 108 months, respectively (range 1–222 months). Follow-up remained slightly shorter in the DTC-HT group (mean 106.9 months, median 102 months, range 12–212 months) than in the DTC-non-HT group (mean 112.4 months, median 109 months, range 1–222 months).

### 3.2. Outcome

During follow-up, 23 of 707 patients (3.25%) developed recurrent disease. At last follow-up, 638 (90.2%) were alive, and 69 (9.8%) had died, including 17 (2.4%) disease-related deaths (2 in the DTC-HT group) and 52 (7.4%) deaths from other causes (3 in the DTC-HT group).

### 3.3. DTC-Non-HT Group

In the DTC-non-HT group, recurrence was documented in 22 of 570 patients (3.9%), whereas 548 (96.1%) remained recurrence-free. Among patients with recurrence, 18 (81.8%) were alive, and 4 (18.2%) were deceased at the time of the last follow-up; including 3 (75%) deaths attributable to thyroid cancer and 1 (25%) due to other causes. Among recurrence-free patients, 488 (89.1%) were alive, and 60 (10.9%) were deceased, including 12 (20.0%) deaths attributable to thyroid cancer and 48 (80.0%) to other causes. Among the surviving recurrence-free patients, 422 (86.5%) achieved a complete response, 21 (4.3%) had a biochemical incomplete response, 15 (3.1%) had a structural incomplete response, and 30 (6.1%) had an indeterminate response.

### 3.4. DTC-HT Group

In the DTC-HT group, recurrence was documented in only 1 of 137 patients (0.7%), whereas 136 patients (99.3%) remained recurrence-free. At the time of the last follow-up, the patient with recurrence was alive, while among recurrence-free patients, 131 (96.3%) were alive and 5 (3.7%) were deceased. Of the 5 deaths, 2 (40.0%) were attributable to thyroid cancer and 3 (60.0%) to other causes. Among the surviving recurrence-free patients, 122 (93.1%) achieved a complete response, 5 (3.8%) had a biochemical incomplete response, 1 (0.8%) had a structural incomplete response, and 3 (2.3%) had an indeterminate response.

### 3.5. Univariate Analysis for DSS and RFS

**Figure 2:**
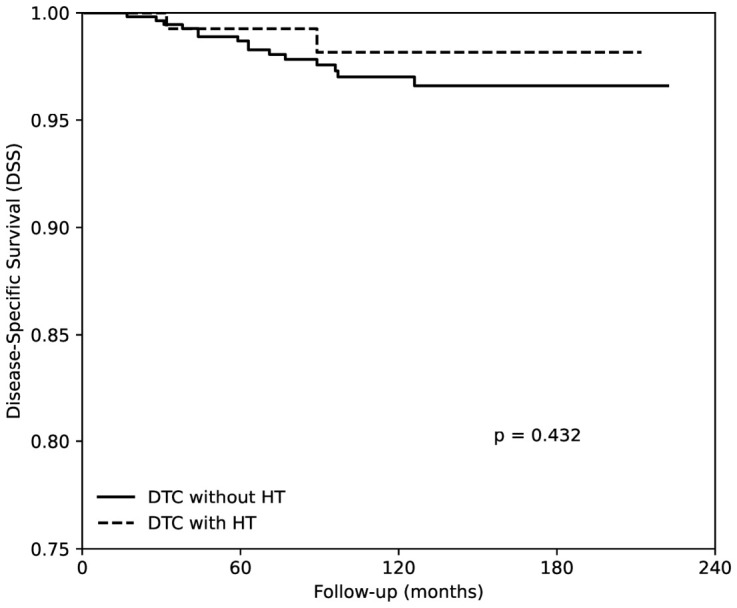
Kaplan–Meier curve of disease-specific survival (DSS) in DTC-HT and DTC-non-HT patients.

Data on DSS were analyzed using Kaplan–Meier curves comparing patients with and without HT. DSS in DTC-HT patients was 99.3% at 5 years and 98.2% at 10, 15, and 20 years. In the DTC-non-HT group, the 5-, 10-, 15-, and 20-year DSS rates were 98.3%, 96.9%, 96.9%, and 96.9%, respectively. No statistically significant difference was detected between the DTC-HT and DTC-non-HT groups (log-rank, *p* = 0.432) (Figure 2).

**Figure 3:**
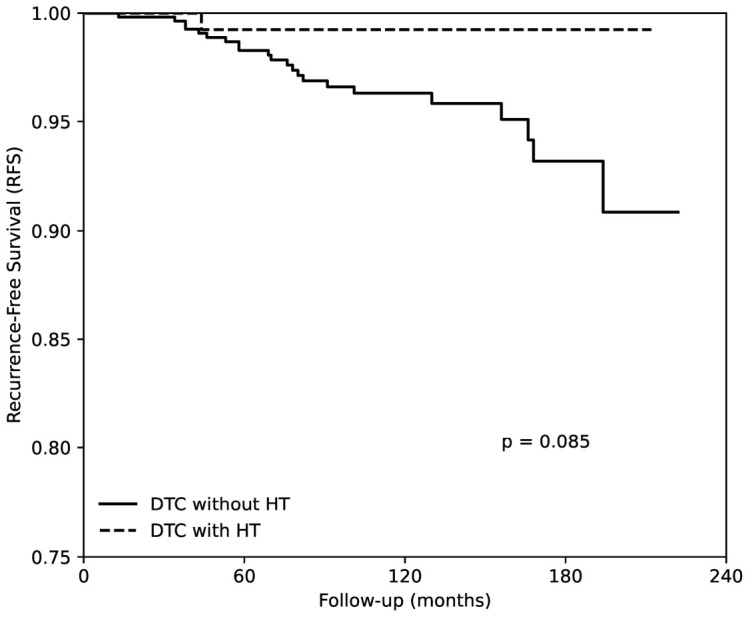
Kaplan–Meier curve of recurrence-free survival (RFS) in DTC-HT patients and DTC-non-HT patients.

Data on RFS were analyzed using a Kaplan–Meier curve comparing RFS in patients with and without HT (Figure 3). RFS in DTC-HT patients was 99.2% at 5, 10, 15, and 20 years. In comparison, RFS rates in DTC-non-HT patients were 98.3%, 96.3%, 93.2%, and 90.9% at 5, 10, 15, and 20 years, respectively. A trend toward reduced RFS was observed in patients without HT compared to those with HT; however, this difference did not reach statistical significance (log-rank, *p* = 0.085) (Figure 3).

### 3.6. Multivariable Analysis for DSS and RFS

Cox proportional hazards regression analysis indicated that HT was not associated with DSS (HR 0.97, 95% CI 0.21–4.52; *p* = 0.964). Similarly, male sex, initial lymph node involvement (N1), and tumor histology were not significantly associated with DSS (HR 1.82, 95% CI 0.59–5.58, *p* = 0.293; HR 1.10, 95% CI 0.32–3.74, *p* = 0.880; HR 0.93, 95% CI 0.25–3.56; *p* = 0.964, respectively).

In contrast, initial distant metastases (M1) and higher pT stage (pT1–2 vs. pT3–4) were independently associated with worse DSS (HR 19.77, 95% CI 6.47–60.42, *p* < 0.001; and HR 11.74, 95% CI 2.41–57.12, *p* = 0.002, respectively). Older age at presentation (≥55 years) showed a borderline association with DSS (HR 3.25, 95% CI 0.99–10.65; *p* = 0.051) (Table 2).

**Figure 4:**
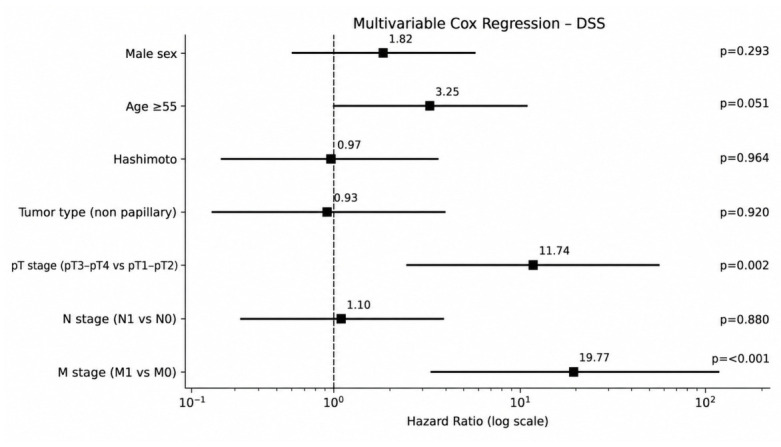
Forest plot of multivariable Cox regression analysis for DSS.

Advanced T stage and distant metastases were independently associated with worse disease-specific survival (Figure 4).

In the multivariable Cox proportional hazards model for RFS, male sex, higher initial higher pT stage (pT3–4 vs. pT1–2), and lymph node involvement at diagnosis were independently associated with reduced RFS (HR 2.98, 95% CI 1.24–7.14, *p* = 0.015; HR 2.97, 95% CI 1.21–7.33, *p* = 0.018; and HR 3.00, 95% CI 1.16–7.73, *p* = 0.023, respectively). In contrast, Hashimoto thyroiditis was not an independent predictor of recurrence (HR 0.36, 95% CI 0.05–2.73, *p* = 0.322). Similarly, tumor histology, older age (≥55 years), and the presence of distant metastases (M1) were not significantly associated with recurrence risk (HR 1.50, 95% CI 0.47–4.76, *p* = 0.489; HR 0.99, 95% CI 0.42–2.30, *p* = 0.977; and HR 1.04, 95% CI 0.13–8.15, *p* = 0.970, respectively) (Table 3).

**Figure 5:**
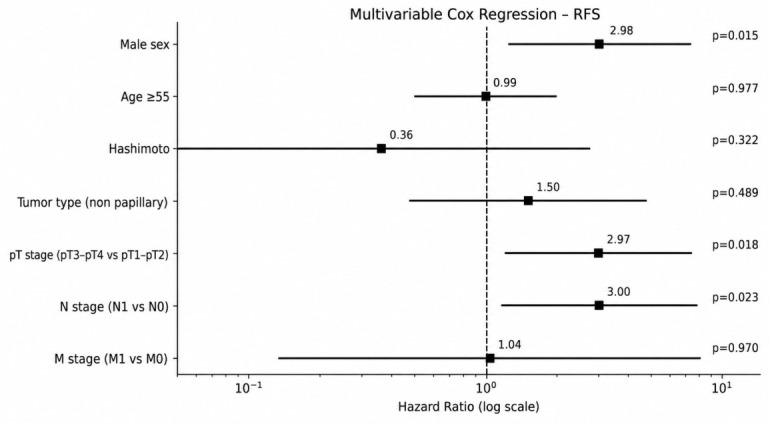
Forest plot of multivariable Cox regression analysis of RFS.

Male sex, advanced T stage, and nodal involvement were detected as independent predictors of recurrence (Figure 5).

## 4. Discussion

In the present study, we evaluated the impact of clinicopathological factors, including HT, on DSS, RFS, and overall clinical outcomes in patients with DTC. In our cohort, HT was present in 19.4% of patients with DTC, and in 18.1% of those with PTC. Our results are consistent with other reports on the prevalence of HT in PTC, ranging from 0.4% to 43.7% [9,14,16,18,21,22,23,24,25,26,27,28,29,30,31,32,33,34,35,36,37,38,39,40,41,42,43,44,45,46,47,48]. However, some authors have reported a much higher frequency of coexistent HT in PTC, reaching 55% [49] and 63% [50].

The link between inflammation and cancer has been recognized since 1863, when Rudolf Virchow first observed leukocytes in tumor tissue and suggested their role in cancer development [51]. Later, in 1955, the association between papillary thyroid carcinoma and chronic inflammation in HT was first described by Dailey et al. [21]. Whether coexisting HT influences the outcome and prognosis of differentiated thyroid cancer remains a matter of debate. Although the available literature is inconsistent and no definitive conclusion has been reached, several studies suggest that HT may have a favorable impact on DTC outcomes. The majority of evidence points toward a potential protective effect of coexistent HT, possibly mediated through immune mechanisms [17,52,53]. The chronic lymphocytic infiltration characteristic of HT may represent an active antitumor immune response, creating a microenvironment capable of limiting tumor progression and metastatic spread [54,55,56,57]. One possible explanation for the observed findings is that a local antitumor immune response directed against the tumor, manifested by peritumoral or intratumoral lymphocytic infiltration, may influence prognosis, whereas concomitant Hashimoto’s thyroiditis as an independent autoimmune condition may not exert the same effect. Tumor-associated lymphocytic infiltration is considered a component of the tumor immune microenvironment and may reflect an active antitumor immune response [58,59]. In contrast, HT represents a chronic autoimmune process characterized by diffuse lymphocytic infiltration, fibrosis, and germinal center formation within the thyroid gland [58,60,61,62].

However, the available literature does not consistently distinguish between these two entities, making it difficult to determine their independent contributions to patient outcomes. Although several studies and meta-analyses have reported more favorable clinicopathological characteristics and outcomes in patients with papillary thyroid carcinoma and coexisting HT, the underlying mechanisms remain incompletely understood [35,56,60,63]. It is therefore possible that at least part of the reported prognostic benefit attributed to HT reflects the influence of tumor-associated immune responses rather than HT itself [61,64]. Further studies specifically evaluating the independent effects of HT and tumor-directed lymphocytic infiltration are needed to clarify their respective prognostic significance.

Cytotoxic T lymphocytes and natural killer cells can recognize and eliminate malignant cells, while thyroid-specific antigens such as thyroglobulin and thyroid peroxidase may serve as targets for immune-mediated cytotoxicity [30,65,66]. In addition, increased apoptosis may contribute to this protective effect. Giordano et al. demonstrated that follicular cells in chronic lymphocytic thyroiditis (CLT) express Fas and Fas ligand, activating programmed cell death pathways that may affect both normal and malignant thyrocytes, potentially resulting in less aggressive tumor behavior [67].

Genetic factors may also contribute to this protective effect. It has been reported that the BRAF V600E mutation is associated with more aggressive clinicopathological features, including extrathyroidal extension, lymph node metastases, advanced TNM stage, and recurrence in PTC [15,68,69,70,71], yet HT is observed less frequently in BRAF V600E-positive tumors [72]. This may partly explain the less aggressive tumor characteristics and more favorable prognosis observed in patients with HT [73,74]. Moreover, in BRAF wild-type DTC, HT has been reported to independently reduce recurrence risk by approximately 70% [15]. Collectively, these findings suggest that immune activation and BRAF mutation status may jointly influence the prognostic impact of autoimmune thyroiditis in thyroid cancer.

Improved disease-free survival (DFS) in some patients may be explained by a higher incidence of microcarcinoma, characterized by smaller tumor size, lower rates of nodal metastasis, and extrathyroidal extension [9,75,76,77]. Although the precise relationship between coexisting HT and micropapillary thyroid carcinoma (MPTC) remains unclear, it has been suggested that HT may contribute to MPTC development by altering the thyroid microenvironment through chronic inflammation [78]. In our study, we similarly found a significantly higher frequency of low initial tumor stage (pT1–pT2) in the DTC-HT group than in the DTC-non-HT group (86.2% vs. 76.1%, *p* = 0.015).

Several authors suggest that DTCs with coexisting HT have reported a higher rate of tumor multifocality in DTCs coexisting with HT [9,17,32,39,42,64,79,80,81]. Although this finding might suggest a less favorable prognosis, studies have shown that the association between HT and multifocal PTC does not adversely affect clinical outcomes and is associated with a lower rate of nodal metastasis [9,17,79]. In contrast, some authors have reported that coexistent lymphocytic thyroiditis (LT) is not associated with multifocality in patients with PTC [38,41,82]. Similarly, we found no association between tumor multifocality and concomitant HT in patients with DTC. We observed a higher proportion of unifocal than multifocal tumors in both the DTC-HT and DTC-non-HT groups (69.3% vs. 30.7% and 71.6% vs. 28.4%, respectively, *p* = 0.672).

The available evidence suggests that DTC coexisting with HT is generally associated with more favorable clinicopathological characteristics, usually including female predominance, smaller tumor size, lower rates of extrathyroidal extension (ETE), fewer lymph node metastases, and earlier TNM stage.

Several authors have suggested a predominance of female sex in DTC patients with coexisting HT [17,30,43]. Similar findings regarding younger age and female predominance were also described by others [36,83]. Tumor characteristics also appear to be less aggressive in the presence of HT. Smaller tumor size has been consistently reported [36,76,83], while Capellacci et al. found a higher prevalence of stage I disease in HT patients [39]. Similarly, Babli reported earlier TNM stage and lower rates of disease persistence in patients with chronic lymphocytic thyroiditis [43].

With respect to local tumor invasiveness, several researchers demonstrated significantly lower rates of ETE in patients with chronic lymphocytic thyroiditis [17,27,34,84]. In addition, Kim et al. observed lower frequencies of lymphovascular invasion, perineural invasion, and initial lymph node metastases in PTC patients with thyroiditis [84]. However, Yoon et al. and Capellacci et al. did not confirm these associations after multivariate adjustment and ultimately reported no significant differences in ETE or lymph node metastasis following adjustment for confounders [36,39]. In contrast, in another study, HT was identified as a negative predictor of central lymph node metastases [85].

Regarding lymph node involvement, HT has been identified as a negative independent predictor of central lymph node metastasis by some authors. Lee et al. detected a significant association between HT and the absence of nodal metastasis [17]. In accordance with this report, Loh et al. found lower rates of nodal involvement in patients with chronic lymphocytic thyroiditis [30]. In contrast, others did not observe significant differences in lymph node metastasis rates after multivariate analysis [36,76]. Our results showed significant differences between the DTC-HT group and the DTC-non-HT group with regard to the higher proportion of females, lower pT stage, and less lymph node involvement at presentation (*p* = 0.007, *p* = 0.015, and *p* = 0.044, respectively). However, the prevalence of distant metastases at presentation did not differ between the two analyzed groups (*p* = 0.915).

There is a large body of evidence supporting the association between PTC-associated HT and lower recurrence and mortality rates compared with PTC without HT. For instance, Kashima et al. reported a disease-specific mortality (DSM) rate of 0.7% and a 10-year RFS of 95% in patients with CLT, compared with 5% mortality and 85% RFS in patients without CLT [27]. Subsequent studies have corroborated these observations [30,38]. In a large cohort of 631 patients with LT, encompassing both Hashimoto’s thyroiditis and peritumoral lymphocytic infiltration, Loh et al. found significantly lower recurrence (6.3% vs. 24.1%; *p* < 0.0001) and DSM rates (0.8% vs. 8.0%; *p* < 0.001) compared with patients without LT [30]. Similarly, Jeong et al. observed higher RFS in patients with CLT (98.46%) than in those without CLT (95.02%) after a mean follow-up of 58 months [38].

Demir et al. reported DSM rates of 4.79% in DTC patients with coexistent HT and 1.43% in PTC patients without HT, although HT was not significantly associated with 10-year RFS (*p* = 0.059) [14]. In a nationwide Korean cohort of 4398 patients with DTC, Yang et al. demonstrated significantly lower overall and cancer-specific mortality in patients with coexistent HT, with HT independently associated with reduced DTC-related mortality (HR 0.33; 95% CI 0.14–0.77) [86]. Xu et al. reported superior unadjusted 10-year DSS (99.9% vs. 96.6%; *p* < 0.001) and RFS (92.0% vs. 87.6%; *p* = 0.001) in patients with HT compared with those without, and multivariable analysis confirmed that HT independently predicted decreased PTC-related mortality [75].

Large retrospective series further support these findings. Huang et al. demonstrated a significantly higher recurrence rate in patients with PTC alone compared with those with coexistent CLT (*p* = 0.0148), with thyroid cancer–specific survival at 10 and 20 years of 95.7% and 91.8% in PTC-only patients versus 98.7% at both time points in PTC-CLT patients [87]. Matsubayashi et al. similarly reported a markedly reduced recurrence rate in tumors with histologic lymphocytic infiltration compared with those without (2.8% vs. 18.6%) [29]. Yang et al. also observed improved RFS in patients with coexistent HT (99.2% vs. 94.5%; *p* = 0.045), an association that remained significant after adjustment for age and sex [83]. Collectively, these and other reports suggest that patients with coexisting Hashimoto’s thyroiditis and PTC exhibit less aggressive tumor features and improved clinical outcomes [16,88,89].

The favorable outcomes of PTC associated with HT have also been confirmed in multiple meta-analyses. Singh et al. reported a positive correlation between HT and both DFS (r = 0.09; 95% CI 0.05–0.12) and overall survival (r = 0.11; 95% CI 0.07–0.15) [90]. Similarly, Moon et al., in a meta-analysis of 71 studies including 44,034 participants (25.3% with HT), demonstrated that coexistent HT was significantly associated with a reduced risk of recurrence (RR 0.50; 95% CI 0.41–0.61) [35]. Lee et al., analyzing 38 studies comprising 10,648 PTC cases, found that histologically confirmed HT (23.2% of cases) was significantly associated with prolonged recurrence-free survival (HR 0.60; *p* = 0.001) [17]. Furthermore, Xu et al., in a meta-analysis of 39 studies, reported a substantially better prognosis in HT-associated carcinomas, with a pooled odds ratio of 0.32 (95% CI 0.18–0.58; *p* = 0.0002) for recurrence [79].

In contrast to previous studies, some literature reports did not find a significant effect of coexistent CLT on the course of PTC [46,80,90,91]. In a multivariate analysis, Kebebew et al. obtained that CLT was not an independent prognostic factor and was not associated with a lower rate of recurrence and distant metastasis [80]. No statistically significant difference between the DTC-HT and DTC-non-HT groups was observed in recurrence rate or overall survival [18,34,39].

Data on the frequency of lymph node metastases in DTCs with concomitant HT and their influence on the outcome are also conflicting. Some authors have reported that the PTC-HT group had better long-term outcomes and a lower frequency of lymph node metastasis than the PTC-non-HT group [9]. HT has been identified as an independent negative predictor of lymph node involvement at presentation [16]. Conversely, some studies suggest a worse prognosis among DTC patients associated with HT [14,41,92,93,94], due to a higher risk of cervical lymph node metastases. Additionally, HT has been associated with aggressive tumor features (such as lymphovascular invasion and nodal metastasis) and lower survival in PTC patients [14].

In a study by Jeong et al., among a subset of 597 patients, the RFS rate in patients with CLT was significantly higher than in those without CLT (*p* = 0.042). However, multivariate analysis found that CLT was not a significant independent negative predictor of recurrence, although its presence was associated with a reduced risk of recurrence [38]. Our results are in alignment with the latter study.

In the present study, patients with DTC and concomitant HT demonstrated more favorable outcomes compared with those without HT. DTC-HT patients had better outcomes than DTC-non-HT patients. During the follow-up period, recurrent disease occurred less frequently in the DTC-HT group than in the DTC-non-HT group (0.7% vs. 3.9%, respectively). At last follow-up, DTC-HT patients showed better treatment responses, with higher rates of complete response (93.1% vs. 86.5%), lower rates of biochemical incomplete response (3.8% vs. 4.3%), structural incomplete response (0.8% vs. 3.1%), and indeterminate response (2.3% vs. 6.1%).

Our results show that although improved RFS was observed in the univariate analysis, HT was not identified as an independent predictor of recurrence after multivariable adjustment (HR 0.36, 95% CI 0.05–2.73; *p* = 0.322). However, male sex, advanced pT stage (pT3–4 vs. pT1–2), and initial lymph node involvement were independently associated with decreased RFS. The favorable association detected in the univariate model may be explained by the more advantageous clinicopathological profile of patients with concomitant HT, particularly a lower pT stage and reduced lymph node involvement at presentation. After controlling for these established prognostic factors, the association between HT and recurrence was no longer statistically significant. These findings suggest that HT is not an independent determinant of recurrence risk but rather a marker associated with less aggressive tumor characteristics at diagnosis. The interpretation of the non-significant difference in recurrence-free survival should, however, remain cautious, given the low number of recurrence events observed during follow-up, which may have limited the statistical power to detect a modest independent effect of HT. In addition, the discrepancy between predictors of RFS and DSS highlights the biological distinction between recurrence and mortality. While locoregional tumor burden (T and N stage) appears to drive recurrence risk, distant metastasis (M stage) remains the dominant determinant of disease-specific mortality. This supports the concept that RFS reflects tumor aggressiveness, whereas DSS reflects ultimate disease lethality.

This study has several limitations that should be acknowledged. First, the generally excellent prognosis of DTC, particularly in a cohort predominantly composed of pT1–2 N0 tumors, inevitably limits the ability to detect small differences in DFS and DSS. Despite the relatively large sample size (707 patients) and long median follow-up (109 months), the relatively low number of recurrence events and disease-specific deaths, particularly in the HT group, may have limited the statistical power to identify subtle prognostic effects of HT. Therefore, although our results do not demonstrate a significant association between HT and survival outcomes, we cannot exclude the possibility of a small effect that would require larger cohorts or pooled analyses to detect. Second, the retrospective design of the study may limit the strength of the evidence and introduce biases related to data collection and patient selection. Third, the assessment of Hashimoto’s thyroiditis was based on histopathological reports and did not include detailed characterization of the tumor immune microenvironment, such as lymphocyte subpopulations, or molecular data, including BRAF mutation status. Finally, validation in independent cohorts is necessary to confirm the reproducibility and generalizability of these findings.

## 5. Conclusions

Although the coexistence of Hashimoto’s thyroiditis and differentiated thyroid cancer in our cohort was associated with more favorable clinicopathological characteristics and improved univariate outcomes, HT was not independently associated with disease-specific survival or recurrence-free survival in multivariable analyses. Given the excellent overall prognosis of this predominantly low-risk DTC population, a modest prognostic effect of HT cannot be entirely excluded. Nevertheless, our findings suggest that any potential impact of HT is likely modest compared with established clinicopathological risk factors, particularly tumor stage. Conventional staging parameters, especially T and M stage, remain the principal determinants of recurrence and survival in DTC. Future studies incorporating detailed immune profiling and molecular biomarkers, ideally in larger multicenter cohorts, may further refine risk stratification and support more personalized patient management.

## Figures and Tables

**Table 1:** Demographic and clinico-pathological patients’ data.

Variable	DTC-HT (*n* = 137)	DTC-Non-HT (*n* = 570)	*p*-Value
Sex			
Female	124 (90.5%)	458 (80.4%)	0.007
Male	13 (9.5%)	112 (19.6%)	
Age at diagnosis			
≤55 years	82 (59.9%)	313 (54.9%)	0.342
≥55 years	55 (40.1%)	257 (45.1%)	
Histological type			
Papillary	128 (93.4%)	500 (87.7%)	0.079 *
Follicular	6 (4.4%)	54 (9.5%)	
Oncocytic	3 (2.2%)	16 (2.8%)	
Tumor focality			
Unifocal	95 (69.3%)	408 (71.6%)	0.672
Multifocal	42 (30.7%)	162 (28.4%)	
pT stage			
pT1	99 (72.3%)	320 (56.1%)	0.015 **
pT2	19 (13.9%)	114 (20.0%)	
pT3	19 (13.9%)	127 (22.3%)	
pT4	0 (0%)	9 (1.6%)	
N stage			
N0	125 (91.2%)	479 (84.0%)	0.044
N1	12 (8.8%)	91 (16.0%)	
M stage			
M0	134 (97.8%)	554 (97.2%)	0.915
M1	3 (2.2%)	16 (2.8%)	
Initial treatment			
TT + RAI	95 (69.3%)	397 (69.6%)	0.526
TT − RAI	39 (28.5%)	147 (25.8%)	
STT + RAI	3 (2.2%)	21 (3.7%)	
STT − RAI	0 (0%)	5 (0.9%)	

TT—total thyroidectomy; STT—subtotal thyroidectomy; RAI—radioactive iodine therapy; *—PTC vs. other DTC; **—pT1–2 vs. pT3–4.

**Table 2:** Cox proportional hazards regression analysis—DSS.

Variable	HR	95% CI	*p*-Value
Initial M stage	19.77	6.47–60.42	<0.001
Initial pT stage	11.74	2.41–57.12	0.002
Age ≥55	3.25	0.99–10.65	0.051
Male sex	1.82	0.59–5.58	0.293
Initial N stage	1.10	0.32–3.74	0.880
Tumor type	0.93	0.25–3.56	0.920
HT	0.97	0.21–4.52	0.964

**Table 3:** Cox proportional hazards regression analysis—RFS.

Variable	HR	95% CI	*p*-Value
Male gender	2.98	1.24–7.14	0.015
Initial pT stage	2.97	1.21–7.33	0.018
Initial N stage	3.00	1.16–7.73	0.023
Tumor type	1.50	0.47–4.76	0.489
HT	0.36	0.05–2.73	0.322
Age ≥55	0.99	0.42–2.30	0.977
Initial M stage	1.04	0.13–8.15	0.970

## Data Availability

The data presented in this study are available on request from the corresponding author. The data are not publicly available due to patients’ privacy.

## Abbreviations

The following abbreviations are used in this manuscript:

AJCC	American Joint Committee on Cancer
ATA	American Thyroid Association
CT	Computed Tomography
DSS	Disease-Specific Survival
DTC	Differentiated Thyroid Carcinoma
ETE	Extrathyroidal Extension
FNAC	Fine-Needle Aspiration Cytology
FT3	Free Triiodothyronine
FT4	Free Thyroxine
HT	Hashimoto Thyroiditis
HR	Hazard Ratio
PTC	Papillary Thyroid Carcinoma
RAI	Radioactive Iodine
RFS	Recurrence-Free Survival
Tg	Thyroglobulin
TgAb	Antithyroglobulin Antibodies
TNM	Tumor–Node–Metastasis
TPOAb	Thyroid Peroxidase Antibodies
TSH	Thyroid-Stimulating Hormone
TT	Total Thyroidectomy
US	Ultrasound
WBS	Whole-Body Scintigraphy

## References

[1] Chmielik E., Rusinek D., Oczko-Wojciechowska M., Jarzab M., Krajewska J., Czarniecka A., Jarzab B. (2018). Heterogeneity of Thyroid Cancer. Pathobiology.

[2] Kaliszewski K., Diakowska D., Miciak M., Jurkiewicz K., Kisiel M., Makles S., Dziekiewicz A., Biernat S., Ludwig M., Ludwig B. (2023). The Incidence Trend and Management of Thyroid Cancer—What Has Changed in the Past Years: Own Experience and Literature Review. Cancers.

[3] Cancer Genome Atlas Research Network (2014). Integrated genomic characterization of papillary thyroid carcinoma. Cell.

[4] Zhang J., Xu S. (2024). High aggressiveness of papillary thyroid cancer: From clinical evidence to regulatory cellular networks. Cell Death Discov..

[5] Bray F., Laversanne M., Sung H., Ferlay J., Siegel R.L., Soerjomataram I., Jemal A. (2024). Global cancer statistics 2022: GLOBOCAN estimates of incidence and mortality worldwide for 36 cancers in 185 countries. CA Cancer J. Clin..

[6] Siegel R.L., Kratzer T.B., Wagle N.S., Sung H., Jemal A. (2026). Cancer statistics, 2026. CA Cancer J. Clin..

[7] Tywanek E., Michalak A., Świrska J., Zwolak A. (2024). Autoimmunity, New Potential Biomarkers and the Thyroid Gland—The Perspective of Hashimoto’s Thyroiditis and Its Treatment. Int. J. Mol. Sci..

[8] Almahari S.A., Maki R., Al Teraifi N., Alshaikh S., Chandran N., Taha H. (2023). Hashimoto Thyroiditis beyond Cytology: A Correlation between Cytological, Hormonal, Serological, and Radiological Findings. J. Thyroid Res..

[9] Zhu F., Shen Y.B., Li F.Q., Fang Y., Hu L., Wu Y.J. (2016). The Effects of Hashimoto Thyroiditis on Lymph Node Metastases in Unifocal and Multifocal Papillary Thyroid Carcinoma: A Retrospective Chinese Cohort Study. Medicine.

[10] Jonklaas J. (2022). Optimal Thyroid Hormone Replacement. Endocr. Rev..

[11] Hashimoto H. (1912). Zur Kenntniss der lymphomatosen Verandderung der Schilddruse (Struma Lymphomatosa). Arch. Klin. Chir..

[12] Rose N.R., Witebsky E. (1956). Studies in organ specificity. V. Changes in the thyroid glands of rabbits following active immunization with rabbit thyroid extracts. J. Immunol..

[13] Roitt I.M., Doniach D., Campbell P.N., Hudson R.V. (1956). Auto-antibodies in Hashimoto’s disease (lymphadenoid goitre). Lancet.

[14] Demir A.N., Kara Z., Sulu C., Uysal S., Sahin S., Zulfaliyeva G., Atar O.A., Valikhanova N., Ozturk T., Ozkaya H.M. (2023). Does the Association of Hashimoto’s Thyroiditis with Differentiated Thyroid Cancer Really Have a Protective Role?. Horm. Metab. Res..

[15] Issa P.P., Omar M., Buti Y., Aboueisha M., Munshi R., Hussein M., Haidari M., Blair G., Issa C.P., Shama M. (2023). Hashimoto’s Thyroiditis: A Protective Factor against Recurrence in BRAF-Wild Type Differentiated Thyroid Carcinoma. Cancers.

[16] Dvorkin S., Robenshtok E., Hirsch D., Strenov Y., Shimon I., Benbassat C.A. (2013). Differentiated thyroid cancer is associated with less aggressive disease and better outcome in patients with coexisting Hashimoto’s thyroiditis. J. Clin. Endocrinol. Metab..

[17] Lee J.H., Kim Y., Choi J.W., Kim Y.S. (2013). The association between papillary thyroid carcinoma and histologically proven Hashimoto’s thyroiditis: A meta-analysis. Eur. J. Endocrinol..

[18] Donnici A., Mirabelli M., Giuliano S., Misiti R., Tocci V., Greco M., Aiello V., Brunetti F.S., Chiefari E., Aversa A. (2024). Coexistence of Hashimoto’s Thyroiditis in Differentiated Thyroid Cancer: Post-Operative Monitoring of Anti-Thyroglobulin Antibodies and Assessment of Treatment Response. Diagnostics.

[19] Haugen B.R., Alexander E.K., Bible K.C., Doherty G.M., Mandel S.J., Nikiforov Y.E., Pacini F., Randolph G.W., Sawka A.M., Schlumberger M. (2016). 2015 American Thyroid Association Management Guidelines for Adult Patients with Thyroid Nodules and Differentiated Thyroid Cancer: The American Thyroid Association Guidelines Task Force on Thyroid Nodules and Differentiated Thyroid Cancer. Thyroid.

[20] Brierley J.D., Gospodarowicz M.K., Wittekind C. (2017). TNM Classification of Malignant Tumours.

[21] Dailey M.E., Lindsay S., Skahen R. (1955). Relation of thyroid neoplasms to Hashimoto disease of the thyroid gland. A.M.A. Arch. Surg..

[22] Woolner L.B., McConahey W.M., Beahrs O.H. (1959). Struma lymphomatosa (Hashimoto’s thyroiditis) and related thyroidal disorders. J. Clin. Endocrinol. Metab..

[23] Hirabayashi R.N., Lindsay S. (1965). The relation of thyroid carcinoma and chronic thyroiditis. Surg. Gynecol. Obstet..

[24] Crile G. (1978). Struma lymphomatosa and carcinoma of the thyroid. Surg. Gynecol. Obstet..

[25] Segal K., Ben-Bassat M., Avraham A., Har-El G., Sidi J. (1985). Hashimoto’s thyroiditis and carcinoma of the thyroid gland. Int. Surg..

[26] DeGroot L.J., Kaplan E.L., McCormick M., Straus F.H. (1990). Natural history, treatment, and course of papillary thyroid carcinoma. J. Clin. Endocrinol. Metab..

[27] Kashima K., Yokoyama S., Noguchi S., Murakami N., Yamashita H., Watanabe S., Uchino S., Toda M., Sasaki A., Daa T. (1998). Chronic thyroiditis as a favorable prognostic factor in papillary thyroid carcinoma. Thyroid.

[28] Schäffler A., Palitzsch K.D., Seiffarth C., Höhne H.M., Riedhammer F.J., Hofstädter F., Schölmerich J., Rüschoff J. (1998). Coexistent thyroiditis is associated with lower tumour stage in thyroid carcinoma. Eur. J. Clin. Investig..

[29] Matsubayashi S., Kawai K., Matsumoto Y., Mukuta T., Morita T., Hirai K., Matsuzuka F., Kakudoh K., Kuma K., Tamai H. (1995). The correlation between papillary thyroid carcinoma and lymphocytic infiltration in the thyroid gland. J. Clin. Endocrinol. Metab..

[30] Loh K.C., Greenspan F.S., Dong F., Miller T.R., Yeo P.P. (1999). Influence of lymphocytic thyroiditis on the prognostic outcome of patients with papillary thyroid carcinoma. J. Clin. Endocrinol. Metab..

[31] Matesa-Anić D., Matesa N., Dabelić N., Kusić Z. (2009). Coexistence of papillary carcinoma and Hashimoto’s thyroiditis. Acta Clin. Croat..

[32] Wu K., Shi L., Wang J., Xie L. (2023). Association between papillary thyroid carcinoma and lymphocytic thyroiditis: A retrospective study. Oncol. Lett..

[33] Kim E.Y., Kim W.G., Kim W.B., Kim T.Y., Kim J.M., Ryu J.S., Hong S.J., Gong G., Shong Y.K. (2009). Coexistence of chronic lymphocytic thyroiditis is associated with lower recurrence rates in patients with papillary thyroid carcinoma. Clin. Endocrinol..

[34] Lau J., Lee J., Mahipal M., Yang S.P., Tan W.B., Yuan N.K., Parameswaran R. (2022). Hashimoto’s thyroiditis on outcomes in papillary thyroid cancer revisited: Experience from South East Asia. Ann. R. Coll. Surg. Engl..

[35] Moon S., Chung H.S., Yu J.M., Yoo H.J., Park J.H., Kim D.S., Park Y.J. (2018). Associations between Hashimoto Thyroiditis and Clinical Outcomes of Papillary Thyroid Cancer: A Meta-Analysis of Observational Studies. Endocrinol. Metab..

[36] Yoon Y.H., Kim H.J., Lee J.W., Kim J.M., Koo B.S. (2012). The clinicopathologic differences in papillary thyroid carcinoma with or without co-existing chronic lymphocytic thyroiditis. Eur. Arch. Otorhinolaryngol..

[37] Zhang Y., Ma X.P., Deng F.S., Liu Z.R., Wei H.Q., Wang X.H., Chen H. (2014). The effect of chronic lymphocytic thyroiditis on patients with thyroid cancer. World J. Surg. Oncol..

[38] Jeong J.S., Kim H.K., Lee C.R., Park S., Park J.H., Kang S.W., Jeong J.J., Nam K.H., Chung W.Y., Park C.S. (2012). Coexistence of chronic lymphocytic thyroiditis with papillary thyroid carcinoma: Clinical manifestation and prognostic outcome. J. Korean Med. Sci..

[39] Cappellacci F., Canu G.L., Lai M.L., Lori E., Biancu M., Boi F., Medas F. (2022). Association between Hashimoto thyroiditis and differentiated thyroid cancer: A single-center experience. Front. Oncol..

[40] Campos L.A., Picado S.M., Guimarães A.V., Ribeiro D.A., Dedivitis R.A. (2012). Thyroid papillary carcinoma associated to Hashimoto’s thyroiditis. Braz. J. Otorhinolaryngol..

[41] Harmantepe A.T., Ozdemir K., Bayhan Z., Kocer B. (2024). The Underestimated Impact of Hashimoto Thyroiditis on Thyroid Papillary Carcinoma. Updates Surg..

[42] Hanege F.M., Tuysuz O., Celik S., Sakallıoglu O., Arslan Solmaz O. (2021). Hashimoto’s thyroiditis in papillary thyroid carcinoma: A 22-year study. Acta Otorhinolaryngol. Ital..

[43] Babli S., Payne R.J., Mitmaker E., Rivera J. (2018). Effects of Chronic Lymphocytic Thyroiditis on the Clinicopathological Features of Papillary Thyroid Cancer. Eur. Thyroid J..

[44] Aydoğan B.İ., Mutlu A.B.B., Yüksel S., Güllü S., Emral R., Demir Ö., Şahin M., Gedik V.T., Çorapçıoğlu D., Sak S.D. (2021). The Association of Histologically Proven Chronic Lymphocytic Thyroiditis with Clinicopathological Features, Lymph Node Metastasis, and Recurrence Rates of Differentiated Thyroid Cancer. Endocr. Pathol..

[45] Grigerova M., Kasko M., Mojtova E., Takacsova E., Kralik R., Waczulikova I., Podoba J. (2022). Influence of autoimmune thyroiditis on the prognosis of papillary thyroid carcinoma. Bratisl. Lek. Listy.

[46] Del Rio P., Montana Montana C., Cozzani F., Rossini M., Loderer T., Dall’Aglio E., Cataldo S., Marina M., Graziano C. (2019). Is there a correlation between thyroiditis and thyroid cancer?. Endocrine.

[47] Pradhan R., Goswami C., Sikder M., Mukherjee U., Mondal S. (2024). Association of Hashimoto thyroiditis with papillary thyroid carcinoma and its clinical features—A retrospective study in a tertiary care hospital. Asian J. Med. Sci..

[48] Dobrinja C., Makovac P., Pastoricchio M., Cipolat Mis T., Bernardi S., Fabris B., Piscopello L., de Manzini N. (2016). Coexistence of chronic lymphocytic thyroiditis and papillary thyroid carcinoma. Impact on presentation, management, and outcome. Int. J. Surg..

[49] Danis N., Comlekci A., Yener S., Durak M., Calan M., Solmaz D., Yalcin M.M., Gulcu A., Demir T., Bayraktar F. (2022). Association between Hashimoto’s thyroiditis and papillary thyroid cancer: A single center experience. Acta Endocrinol..

[50] Repplinger D., Bargren A., Zhang Y.W., Adler J.T., Haymart M., Chen H. (2008). Is Hashimoto’s thyroiditis a risk factor for papillary thyroid cancer?. J. Surg. Res..

[51] Balkwill F., Mantovani A. (2001). Inflammation and cancer: Back to Virchow?. Lancet.

[52] Resende de Paiva C., Grønhøj C., Feldt-Rasmussen U., von Buchwald C. (2017). Association between Hashimoto’s Thyroiditis and Thyroid Cancer in 64,628 Patients. Front. Oncol..

[53] Jankovic B., Le K.T., Hershman J.M. (2013). Clinical Review: Hashimoto’s thyroiditis and papillary thyroid carcinoma: Is there a correlation?. J. Clin. Endocrinol. Metab..

[54] Cunha L.L., Morari E.C., Guihen A.C., Razolli D., Gerhard R., Nonogaki S., Soares F.A., Vassallo J., Ward L.S. (2012). Infiltration of a mixture of immune cells may be related to good prognosis in patients with differentiated thyroid carcinoma. Clin. Endocrinol..

[55] French J.D., Weber Z.J., Fretwell D.L., Said S., Klopper J.P., Haugen B.R. (2010). Tumor-associated lymphocytes and increased FoxP3+ regulatory T cell frequency correlate with more aggressive papillary thyroid cancer. J. Clin. Endocrinol. Metab..

[56] Ma H., Li G., Huo D., Su Y., Jin Q., Lu Y., Sun Y., Zhang D., Chen X. (2024). Impact of Hashimoto’s thyroiditis on the tumor microenvironment in papillary thyroid cancer: Insights from single-cell analysis. Front. Endocrinol..

[57] Ma C., Xu J., Zheng G., Liu L., Song X., Zheng H. (2025). Hashimoto’s Thyroiditis Shows Sex- and Age-Dependent Inverse Associations with Papillary Thyroid Carcinoma Progression: A Propensity Score-Matched Analysis of 6963 Surgical Cases. Ann. Surg. Oncol..

[58] Ahn D., Heo S.J., Park J.H., Kim J.H., Sohn J.H., Park J.Y., Park S.K., Park J. (2011). Clinical relationship between Hashimoto’s thyroiditis and papillary thyroid cancer. Acta Oncol..

[59] Wang L., Li W., Ye H., Niu L. (2018). Impact of Hashimotoas thyroiditis on clinicopathologic features of papillary thyroid carcinoma associated with infiltration of tumor-infiltrating lymphocytes. Int. J. Clin. Exp. Pathol..

[60] Kim H.G., Kim E.K., Han K.H., Kim H., Kwak J.Y. (2014). Pathologic spectrum of lymphocytic infiltration and recurrence of papillary thyroid carcinoma. Yonsei Med. J..

[61] Sulaieva O., Chernenko O., Selesnov O., Nechay O., Maievskyi O., Falalyeyeva T., Kobyliak N., Tsyryuk O., Penchuk Y., Shapochka D. (2020). Mechanisms of the Impact of Hashimoto Thyroiditis on Papillary Thyroid Carcinoma Progression: Relationship with the Tumor Immune Microenvironment. Endocrinol. Metab..

[62] Villagelin D.G., Santos R.B., Romaldini J.H. (2011). Is diffuse and peritumoral lymphocyte infiltration in papillary thyroid cancer a marker of good prognosis?. J. Endocrinol. Investig..

[63] Osborne D., Choudhary R., Vyas A., Kampa P., Abbas L.F., Chigurupati H.D., Alfonso M. (2022). Hashimoto’s Thyroiditis Effects on Papillary Thyroid Carcinoma Outcomes: A Systematic Review. Cureus.

[64] Rho M.H., Kim D.W., Hong H.P., Park Y.M., Kwon M.J., Jung S.J., Kim Y.W., Kang T. (2012). Diagnostic value of antithyroid peroxidase antibody for incidental autoimmune thyroiditis based on histopathologic results. Endocrine.

[65] Ehlers M., Schott M. (2014). Hashimoto’s thyroiditis and papillary thyroid cancer: Are they immunologically linked?. Trends Endocrinol. Metab..

[66] Latrofa F., Ricci D., Grasso L., Vitti P., Masserini L., Basolo F., Ugolini C., Mascia G., Lucacchini A., Pinchera A. (2008). Characterization of thyroglobulin epitopes in patients with autoimmune and non-autoimmune thyroid diseases using recombinant human monoclonal thyroglobulin autoantibodies. J. Clin. Endocrinol. Metab..

[67] Giordano C., Stassi G., De Maria R., Todaro M., Richiusa P., Papoff G., Ruberti G., Bagnasco M., Testi R., Galluzzo A. (1997). Potential involvement of Fas and its ligand in the pathogenesis of Hashimoto’s thyroiditis. Science.

[68] Rodolico V., Cabibi D., Pizzolanti G., Richiusa P., Gebbia N., Martorana A., Russo A., Amato M.C., Galluzzo A., Giordano C. (2007). BRAF V600E mutation and p27 kip1 expression in papillary carcinomas of the thyroid ≤1 cm and their paired lymph node metastases. Cancer.

[69] Xing M., Clark D., Guan H., Ji M., Dackiw A., Carson K.A., Kim M., Tufaro A., Ladenson P., Zeiger M. (2009). BRAF mutation testing of thyroid fine-needle aspiration biopsy specimens for preoperative risk stratification in papillary thyroid cancer. J. Clin. Oncol..

[70] Lin K.L., Wang O.C., Zhang X.H., Dai X.X., Hu X.Q., Qu J.M. (2010). The BRAF mutation is predictive of aggressive clinicopathological characteristics in papillary thyroid microcarcinoma. Ann. Surg. Oncol..

[71] Li P., Liu Y., Wei T., Wang X., Zhu J., Yang R., Gong Y., Zhao W. (2024). Effect and Interactions of BRAF on Lymph Node Metastasis in Papillary Thyroid Carcinoma With Hashimoto Thyroiditis. J. Clin. Endocrinol. Metab..

[72] Molnár C., Molnár S., Bedekovics J., Mokánszki A., Győry F., Nagy E., Méhes G. (2019). Thyroid Carcinoma Coexisting with Hashimoto’s Thyreoiditis: Clinicopathological and Molecular Characteristics Clue up Pathogenesis. Pathol. Oncol. Res..

[73] Zhang Q., Liu S.Z., Zhang Q., Guan Y.X., Chen Q.J., Zhu Q.Y. (2016). Meta-Analyses of Association Between BRAF(V600E) Mutation and Clinicopathological Features of Papillary Thyroid Carcinoma. Cell. Physiol. Biochem..

[74] Zeng R.C., Jin L.P., Chen E.D., Dong S.Y., Cai Y.F., Huang G.L., Li Q., Jin C., Zhang X.H., Wang O.C. (2016). Potential relationship between Hashimoto’s thyroiditis and BRAF(V600E) mutation status in papillary thyroid cancer. Head Neck.

[75] Xu S., Huang H., Qian J., Liu Y., Huang Y., Wang X., Liu S., Xu Z., Liu J. (2021). Prevalence of Hashimoto Thyroiditis in Adults With Papillary Thyroid Cancer and Its Association With Cancer Recurrence and Outcomes. JAMA Netw. Open.

[76] Wang L., Chen J., Yuan X., Wang J., Sun L., Jiang J., Zhang L., Liu M., Zhou Q. (2022). Lymph node metastasis of papillary thyroid carcinoma in the context of Hashimoto’s thyroiditis. BMC Endocr. Disord..

[77] Uhliarova B., Hajtman A. (2018). Hashimoto’s thyroiditis—An independent risk factor for papillary carcinoma. Braz. J. Otorhinolaryngol..

[78] Muzza M., Degl’Innocenti D., Colombo C., Perrino M., Ravasi E., Rossi S., Cirello V., Beck-Peccoz P., Borrello M.G., Fugazzola L. (2010). The tight relationship between papillary thyroid cancer, autoimmunity and inflammation: Clinical and molecular studies. Clin. Endocrinol..

[79] Xu J., Ding K., Mu L., Huang J., Ye F., Peng Y., Guo C., Ren C. (2022). Hashimoto’s Thyroiditis: A “Double-Edged Sword” in Thyroid Carcinoma. Front. Endocrinol..

[80] Kebebew E., Treseler P.A., Ituarte P.H., Clark O.H. (2001). Coexisting chronic lymphocytic thyroiditis and papillary thyroid cancer revisited. World J. Surg..

[81] Liang J., Zeng W., Fang F., Yu T., Zhao Y., Fan X., Guo N., Gao X. (2017). Clinical analysis of Hashimoto thyroiditis coexistent with papillary thyroid cancer in 1392 patients. Acta Otorhinolaryngol. Ital..

[82] Zhang Y., Dai J., Wu T., Yang N., Yin Z. (2014). The study of the coexistence of Hashimoto’s thyroiditis with papillary thyroid carcinoma. J. Cancer Res. Clin. Oncol..

[83] Yang Y., Liu J., Shi X., Wang M. (2023). Clinical and Pathological Characteristics of Patients With Papillary Thyroid Carcinoma Coexisting With Hashimoto’s Thyroiditis: A Retrospective Cohort Study. Cancer Control.

[84] Kim Y.S., Choi H.J., Kim E.S. (2013). Papillary thyroid carcinoma with thyroiditis: Lymph node metastasis, complications. J. Korean Surg. Soc..

[85] Kim S.S., Lee B.J., Lee J.C., Kim S.J., Jeon Y.K., Kim M.R., Huh J.E., Mok J.Y., Kim B.H., Kim Y.K. (2011). Coexistence of Hashimoto’s thyroiditis with papillary thyroid carcinoma: The influence of lymph node metastasis. Head. Neck.

[86] Yang I., Yu J.M., Chung H.S., Kim Y.J., Roh Y.K., Choi M.K., Park S.H., Park Y.J., Moon S. (2024). Hashimoto Thyroiditis and Mortality in Patients with Differentiated Thyroid Cancer: The National Epidemiologic Survey of Thyroid Cancer in Korea and Meta-Analysis. Endocrinol. Metab..

[87] Huang B.Y., Hseuh C., Chao T.C., Lin K.J., Lin J.D. (2011). Well-differentiated thyroid carcinoma with concomitant Hashimoto’s thyroiditis present with less aggressive clinical stage and low recurrence. Endocr. Pathol..

[88] Girardi F.M., Barra M.B., Zettler C.G. (2015). Papillary thyroid carcinoma: Does the association with Hashimoto’s thyroiditis affect the clinicopathological characteristics of the disease?. Braz. J. Otorhinolaryngol..

[89] Kim H.S., Choi Y.J., Yun J.S. (2010). Features of papillary thyroid microcarcinoma in the presence and absence of lymphocytic thyroiditis. Endocr. Pathol..

[90] Singh B., Shaha A.R., Trivedi H., Carew J.F., Poluri A., Shah J.P. (1999). Coexistent Hashimoto’s thyroiditis with papillary thyroid carcinoma: Impact on presentation, management, and outcome. Surgery.

[91] Chesky V.E., Hellwig C.A., Welch J.W. (1962). Cancer of the thyroid associated with Hashimoto’s disease: An analysis of forty-eight cases. Am. Surg..

[92] Vasileiadis I., Boutzios G., Charitoudis G., Koukoulioti E., Karatzas T. (2014). Thyroglobulin antibodies could be a potential predictive marker for papillary thyroid carcinoma. Ann. Surg. Oncol..

[93] Shen C.T., Zhang X.Y., Qiu Z.L., Sun Z.K., Wei W.J., Song H.J., Luo Q.Y. (2017). Thyroid autoimmune antibodies in patients with papillary thyroid carcinoma: A double-edged sword?. Endocrine.

[94] Marongiu A., Nuvoli S., De Vito A., Vargiu S., Spanu A., Madeddu G. (2023). Hashimoto’s thyroiditis and papillary thyroid carcinoma: A follow-up study in patients with absence of aggressive risk factors at the surgery of the primary tumor. Diagnostics.

